# Development of five digits is controlled by a bipartite long-range *cis*-regulator

**DOI:** 10.1242/dev.095430

**Published:** 2014-04

**Authors:** Laura A. Lettice, Iain Williamson, Paul S. Devenney, Fiona Kilanowski, Julia Dorin, Robert E. Hill

**Affiliations:** MRC-Human Genetics Unit, MRC Institute of Genetics and Molecular Medicine, University of Edinburgh, Western General Hospital, Crewe Rd, Edinburgh EH4 2XU, UK

**Keywords:** Sonic hedgehog, Long-range regulation, ZRS, Limb development, Mouse

## Abstract

Conservation within intergenic DNA often highlights regulatory elements that control gene expression from a long range. How conservation within a single element relates to regulatory information and how internal composition relates to function is unknown. Here, we examine the structural features of the highly conserved ZRS (also called MFCS1) *cis*-regulator responsible for the spatiotemporal control of *Shh* in the limb bud. By systematically dissecting the ZRS, both in transgenic assays and within in the endogenous locus, we show that the ZRS is, in effect, composed of two distinct domains of activity: one domain directs spatiotemporal activity but functions predominantly from a short range, whereas a second domain is required to promote long-range activity. We show further that these two domains encode activities that are highly integrated and that the second domain is crucial in promoting the chromosomal conformational changes correlated with gene activity. During limb bud development, these activities encoded by the ZRS are interpreted differently by the fore limbs and the hind limbs; in the absence of the second domain there is no *Shh* activity in the fore limb, and in the hind limb low levels of *Shh* lead to a variant digit pattern ranging from two to four digits. Hence, in the embryo, the second domain stabilises the developmental programme providing a buffer for SHH morphogen activity and this ensures that five digits form in both sets of limbs.

## INTRODUCTION

Multi-species conserved non-coding elements occur in the vertebrate genome and are clustered often within large gene deserts in the vicinity of developmentally regulated genes (Boffelli et al., 2004; Woolfe et al., 2005). Many act as *cis*-regulators of transcription and may reside at long distances from the genes they regulate (Visel et al., 2009). A highly conserved 780-bp element called the ZRS is an example of this class of *cis-*regulator. ZRS sits in an intron of the ubiquitously expressed *Lmbr1* gene and from here it operates over a distance of 1 Mb of DNA to control precisely the spatiotemporal expression of the *Shh* gene in both the fore and hind limbs (Lettice et al., 2002, 2003; Sagai et al., 2005). SHH is a morphogen that is produced in a single, restricted domain lying at the posterior margin of the developing limb bud called the zone of polarising activity (ZPA).

**Fig. 1.**
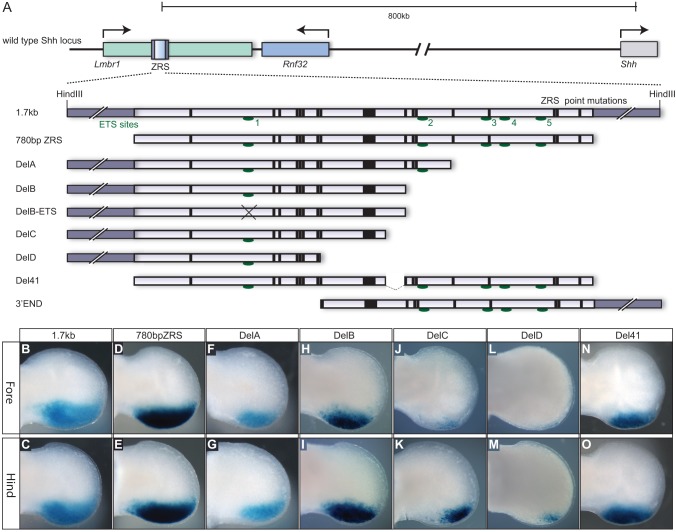
**Deletion analysis of the ZRS *cis*-regulator.** (A) Diagram of the *Shh* locus including the upstream gene desert and the position of the ZRS within an intron of the *Lmbr1* gene. An expanded view of the 1.7-kb *Hin*dIII genomic fragment containing the 780-bp highly conserved ZRS is shown with the position of the point mutations (bars) and the ETS binding sites (green ovals) marked. The deletion constructs used in the transgenic analysis are shown below. (B-O) Expression of the *lacZ* gene in E11.5 embryonic limb buds (fore limbs in the top row and hind limbs in the bottom panel) for the 1.7-kb wild-type fragment (B,C), the conserved 780-bp ZRS (D,E), the DelA deletion (F,G), the DelB deletion (H,I), the DelC deletion (J,K), the DelD deletion (L,M) and the Del41 deletion (N,O). No expression was observed for the DelB-ETS or the 3′ END construct.

The precise spatiotemporal expression of *Shh* in the limb bud is perturbed in response to mutations within the ZRS. Mutations cause a spectrum of limb abnormalities called the ‘ZRS-associated syndromes’, which include preaxial polydactyly type II (PPD2), triphalangeal thumb polysyndactyly (TPTPS), syndactyly type IV (SD4) and Werners mesomelic syndrome (WMS) (for review, see Anderson et al., 2012). Point mutations at >20 different sites in the ZRS (Fig. 1A) cause limb deformities by misdirecting *Shh* expression to an additional, ectopic site located along the anterior margin of the limb. Transgenic mouse assays have proven to be particularly robust (Maas and Fallon, 2005; Masuya et al., 2006; Furniss et al., 2008; Lettice et al., 2008) as a means for measuring the spatial expression activity of both the wild-type ZRS *cis*-regulator and its mutated versions.

A central feature of enhancers is their ability to function as transcription factor-binding platforms that contain a clustering of binding sites for the formation of large protein-DNA complexes (Buecker and Wysocka, 2012). The high degree of conservation found in many long-range enhancer sequences suggests that there is strong selection for both the group of transcription factors that operate at the regulator and the order and arrangement in which these bind. Transcription factors recognise short degenerate sequences on average 5-12 bp in length; we argue, therefore, that the long stretches of absolute sequence similarity within the ZRS (>85% similarity between human and chick) indicate that more complex rules affecting properties other than simple binding affinity of transcription factors are involved in controlling enhancer activity and functional outcome. How the information encoded by the sequence is interpreted is not well understood. Here, we dissect the activities that reside within the ZRS to investigate the sequence components that are important for regulatory activity and then focus on how these components integrate to convey activity across a long genomic distance.

## RESULTS

### Spatiotemporal information resides in the 5′ half of the ZRS

The regulatory activity that directs limb-specific expression of *Shh* is contained within a 1.7-kb *Hin*dIII fragment (Lettice et al., 2003) (Fig. 1A). Constructs containing this element express the *lacZ* reporter gene in the mesenchyme at the posterior margin of the limb bud in transgenic mice, reflecting the endogenous *Shh* pattern (Fig. 1B,C). The expression activity is, however, confined to the highly conserved ∼780-bp fragment (Fig. 1A,D,E) (Lettice et al., 2012) (the ratio of expressing to total transgenic embryos for each construct is listed in Table 1). In order to dissect the ZRS further, a series of terminal deletions from the 3′ end of the 1.7-kb fragment were made (orientation of the ZRS defined relative to the 5′ end of *Shh*) (Fig. 1A) and analysed for spatial patterns of expression in transgenic embryos harvested at approximately embryonic day (E) 11.5. The constructs called DelA and DelB deleted 232 bp and an additional 74 bp (Fig. 1A), respectively; both showed expression at appreciable levels in the posterior margin of the limb bud (Fig. 1F-I). We previously showed that members of the ETS transcription factor family function at the ZRS to establish and restrict the boundary of *Shh* expression to the posterior margin of the limb (Lettice et al., 2012). GABPα and ETS1 bind to multiple sites, in particular two high affinity sites (sites 1 and 3 shown on Fig. 1A) to regulate the position of the expression boundary and at least one of these high affinity sites is required for reporter gene expression. The DelB transgenic construct removed all but site 1 and, accordingly, the *lacZ* reporter gene was expressed in transgenic embryos (Fig. 1H,I); whereas further modification to specifically mutate the remaining ETS site (the consensus ETS binding site **AGG**AAGT at site 1 was converted to **GCC**AAGT inDelB-ETS, Fig. 1A) (Lettice et al., 2012) showed no detectable expression (Table 1).

**Table 1.**
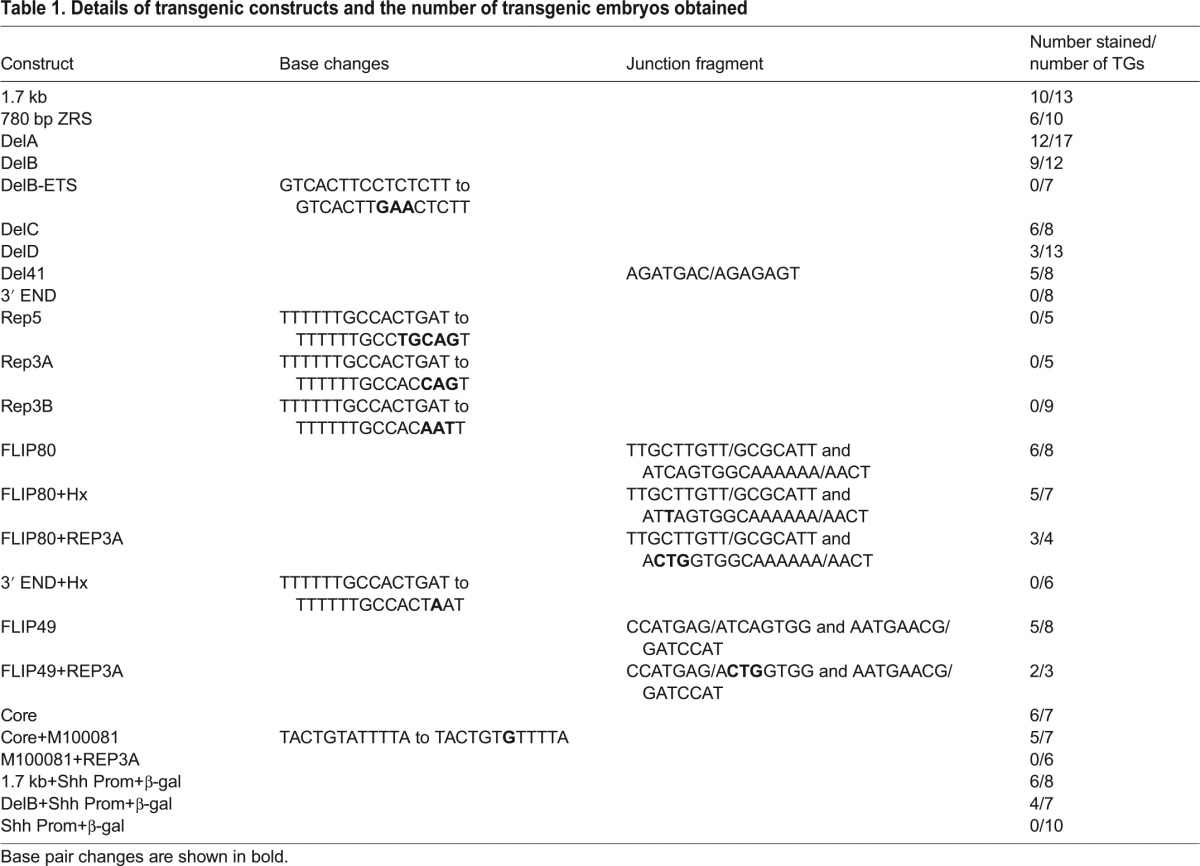
**Details of transgenic constructs and the number of transgenic embryos obtained**

Further deletions caused substantial reductions in the spatial expression pattern as exhibited by constructs DelC and DelD (Fig. 1J-M), which removed an additional 41 bp and 98 bp, respectively (Fig. 1A). The DelC construct revealed lower limb expression and the fore limbs were more susceptible than the hind limbs to this loss of sequence (Fig. 1J,K), suggesting that a forelimb regulatory element lies within the 41-bp fragment between the DelB and DelC deletions. However, the 41-bp sequence was specifically deleted from the intact ZRS (Del41; Fig. 1A) and showed no reduction in expression (Fig. 1N,O), compared with DelB, in either the fore or hind limbs. The final terminal deletion (DelD construct in Fig. 1A) caused a complete loss of forelimb expression, a substantial decrease in the hind limb expression (Fig. 1M) and, overall, a reduction in the percentage of expressing embryos (Table 1). The contribution of the 3′ half of the ZRS, using the 3′END fragment (equivalent to the sequence deleted in DelD in Fig. 1A) was examined but no detectable limb expression (Table 1) was observed, suggesting that this half of the ZRS carries no independent spatial activity. These data indicate that the spatial activity lies in the 5′ half of the ZRS but the activity relies on an accumulative input from throughout the ZRS. Notably, these analyses also showed that the fore and hind limbs respond differently to the loss of sequence information, the fore limbs being more susceptible to this loss.

### ZRS activity requires integration of sequence information

**Fig. 2.**
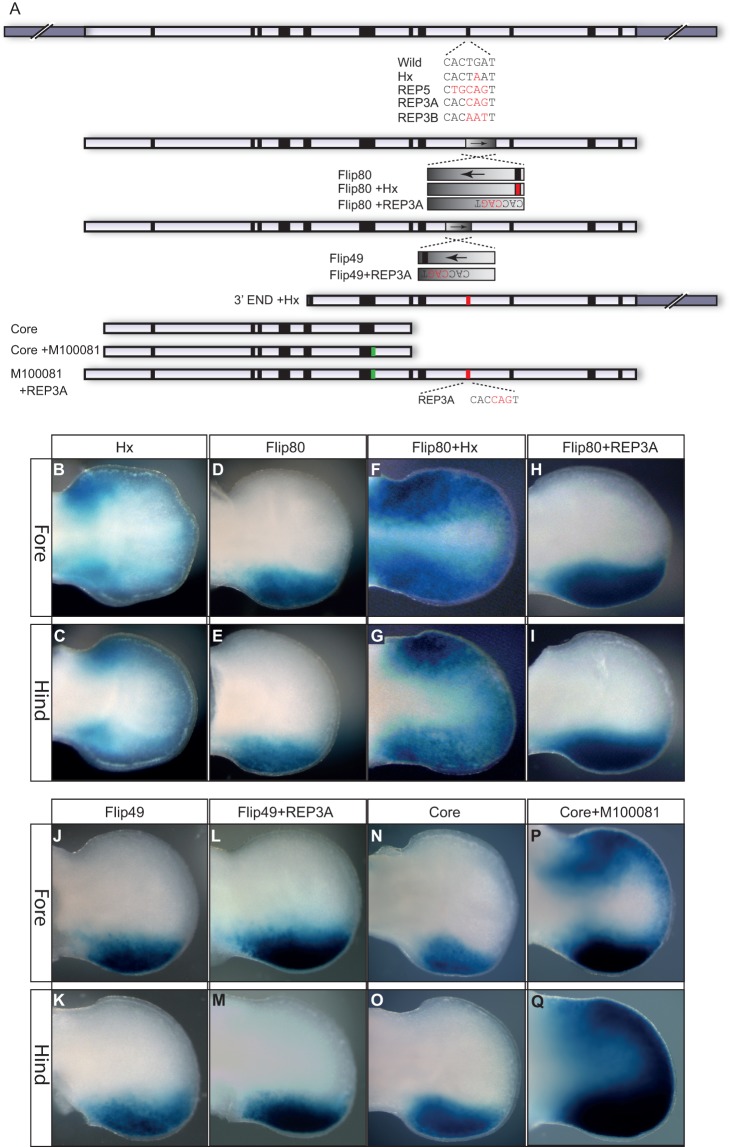
**Mutational analysis within the ZRS.** (A) The modified ZRS constructs used in transgenic analyses. The positions of the point mutations are marked by bars and M100081 and Hx highlighted in green and red, respectively. The sequence changes made within the Hx domain are also shown in red. (B-Q) Expression of the *lacZ* gene in E11.5 embryonic limb buds (fore limbs in the top row of each and hind limbs below) for the construct carrying the *Hx* mutation (B,C) (data from Lettice et al., 2008), the Flip80 construct (D,E), the Flip80+Hx construct (F,G), the Flip80+REP3A construct (H,I), the Flip49 construct (J,K), the Flip49+REP3A (L,M), the Core fragment (N,O) and the Core fragment carrying the M100081 mutation (P,Q). No expression was observed for the 3′END+Hx, the REP3A, REP3B, REP5 or the M100081+REP3A constructs.

Although the 3′ END construct directs no detectable transgenic activity, a number of point mutations associated with preaxial polydactyly reside in this region (Fig. 1A) (Anderson et al., 2012). One well-studied polydactylous mouse mutant, called hemimelic extra toes (*Hx*) (Fig. 2A), is responsible for *Shh* ectopic expression in limb buds (Blanc et al., 2002; Lettice et al., 2003). Accordingly, mouse transgenics that incorporate the *Hx* mutation direct expression (Maas and Fallon, 2005; Lettice et al., 2008) that reflects this mutant pattern (Fig. 2B,C). The deletion series reported above removed the domain containing the *Hx* point mutation but no ectopic expression was detected, indicating that the *Hx* point change acts as a gain-of-function mutation, perhaps generating a novel factor binding site similar to that shown for the AUS and Family AC mutations (Lettice et al., 2012). If the *Hx* mutation generates a novel binding site for an as-yet-unidentified factor then, alternatively, mutations designed to disrupt binding at the *Hx* site specifically should act similarly to the deletions and generate only posterior expression. Nucleotide substitutions at the *Hx* site that should alter binding, a 5-bp replacement (REP5) and two different 3-bp replacements (REP3A and REP3B) (collectively called the REP mutations) (Fig. 2A), all unexpectedly inactivated the ZRS (Table 1). This local disruption of potential factor binding at the *Hx* site had more severe consequences than did the terminal deletions, suggesting that overall structural architecture plays a crucial role in this region of the ZRS.

We predict that the REP mutations disrupt factor binding such that context-dependent protein interactions are altered leading to local inactivation of this region. Terminal deletions, by removing all the regulatory information, bypass these local structural changes such that activity becomes solely dependent on information encoded in the 5′ half of the ZRS. To investigate these local interactions further, the relative position of the *Hx* and REP mutations within the ZRS were rearranged. A gain-of-function *Hx* mutation should be context independent such that the *Hx* mutation site placed anywhere within the ZRS would show ectopic activity. The REP mutations, by contrast, should be highly dependent on the relative position in the sequence. A series of internal inversions were made to test these predictions. An 80-bp highly conserved region was inverted within the context of the intact ZRS such that the encoded information of the inverted 80 bp is retained but situated in the opposite orientation. This construct was called the Flip80 construct (Fig. 2A). Transgenic embryos carrying Flip80 exhibited expression along the wild-type posterior margin (Fig. 2D,E). By contrast, ectopic, anterior expression was detected (Fig. 2F,G) in Flip80 carrying the *Hx* mutation (Flip80+Hx, Fig. 2A) showing that the *Hx*-mediated ectopic expression is not context dependent, consistent with *Hx* acting as a dominant gain-of-function mutation. Next, we made the inversion construct carrying the inactivating REP3A mutation, called Flip80+REP3A (Fig. 2A). Transgenic embryos carrying the REP3A mutation in this context, rather than inactivating the ZRS, showed restoration of expression in the posterior margin (Fig. 2H,I). To assess whether at the inversion breakpoints crucial factor binding sites may have been disrupted, we made another series of inversion constructs (Fig. 2A) called Flip49 and Flip49+REP3A. Both of these exhibited solely posterior expression of the reporter gene (Fig. 2J-M). similar to the Flip80 series. Hence, we suggest that the ZRS activity is not simply a clustering of binding sites but has a structural component key to its activity. Accordingly, the terminal deletions appear to remove sufficient sequence at the 3′ end such that only the spatial information of the ZRS (residing mostly at the 5′ end) is available in the transgenic assay. The REP mutations, by contrast, cause local structural perturbations that affect the whole of the ZRS acting in the manner of an enhanceosome (Panne, 2008). The enhanceosome model suggests that the DNA-protein complex affects structural architecture essential for regulatory activity and, in accordance, the REP mutations underscore the role that local structural integrity plays in affecting the overall activity of the ZRS regulator.

### The point mutations re-direct spatial activity

Because the gain-of-function *Hx* mutation lies within the 3′ half of the ZRS, we examined the ability of this mutation to impart autonomous ectopic activity. The *Hx* point change was incorporated into a construct containing the 3′ END fragment (3′END+Hx, Fig. 2A). The transgenic embryos showed no expression at any site in the embryo (Table 1), suggesting dependence on further information, presumably from the 5′ domain of the ZRS.

To examine further the sequence requirements for driving ectopic expression, we focused on the mouse M100081 mutation (Masuya et al., 2006). The minimal sequence tested that efficiently directed spatial expression in the limb pattern is the Core 443-bp fragment (Fig. 2N,O); created by deleting within the conserved sequence of the ZRS 24 bp from the 5′ end of DelB (Fig. 2A). The M100081 mutation was added to the Core element (Core+M100081, Fig. 2A) and expression was detected, as expected, in the posterior margin but the mutation redirected the expression domain to the anterior of the limb bud (Fig. 2P,Q). To further show that the ectopic expression is dependent on the posterior spatial activity, we added the M100081 mutation to the intact ZRS [transgenic expression previously shown by Lettice et al. (Lettice et al., 2008)] and the ZRS carrying the inactivating REP3 mutation (M100081+REP3A). In the resulting M100081+REP3A transgenic embryos, both the posterior expression and the ectopic activity were undetectable (Table 1). Both the *Hx* and M100081 polydactylous mutations required the spatial information encoded in the 5′ half of the ZRS to operate and, hence, the point mutations function by redirecting expression to the additional, ectopic site in the limb bud. This further supports the notion that the information in the 3′ half is conveyed along the ZRS to affect spatial transcriptional activity encoded in the 5′ half.

### 3′ end of the ZRS required for long-range activity

**Fig. 3.**
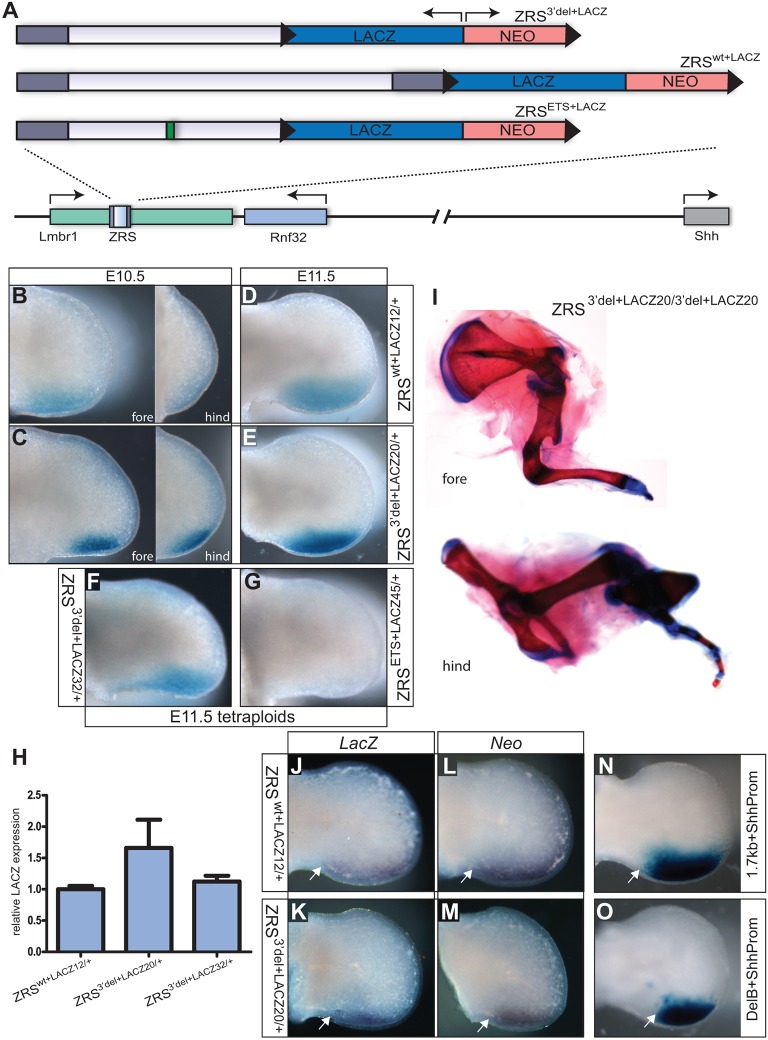
**Analysis of the targeted ZRS locus.** (A) The three targeting constructs used to replace the endogenous ZRS. Each contains the *lacZ* gene (in the opposite orientation to the direction of *Lmbr1* transcription) and the neo^R^ (NEO) gene surrounded by LoxP sites (black triangles). The ZRS^wt+LACZ^ targeted allele contains the 1.7-kb wild-type ZRS fragment. The ZRS^3′del+LACZ^ allele contains a deletion of the 3′ end, retaining the DelB fragment and the ZRS^ETS+LACZ^ allele carries the additional mutation at the ETS1/GABPα binding site (represented by the green rectangle). (B-E) Limb expression of the *lacZ* reporter gene in the following mouse lines: the wild-type line ZRS^wt+LACZ12^ (B,D) and the 3′ deletion line ZRS^3′del+LACZ20^ (C,E). (B,C) ∼E10.5 limb buds, with fore limbs in the left panel and hind limbs in the right panel. (D,E) E11.5 hind limbs. (F,G) The analysis in tetraploid complementation embryos of *lacZ* expression in limb buds from the ZRS^3′del+LACZ32^ (F) and in ZRS^ETS+LACZ45^ limb buds (G). (H) Graph showing the analysis by qRT-PCR of the levels of *lacZ* RNA present in the limb buds of ZRS^wt+LACZ12^ and the two 3′ deletion lines ZRS^3′del+LACZ20^ and ZRS^3′del l+LACZ32^. Mean±s.e.m. (I) Mutant limbs from an E17.5 embryo homozygous for ZRS^3′del+LACZ20/3′del+LACZ20^ showing the severe distal truncations in the fore limbs (upper) and hind limbs (lower). (J-O) The effect of different promoters on expression. (J,K) *In situ* expression of *lacZ* (driven by a β-globin minimal promoter). (L,M) Expression of *NeoR* (driven by the PGK promoter). (J,L) Limbs from the wild-type line ZRS^wt+LACZ12^. (K,M) Limbs from ZRS^3′del l+LACZ20^. (N,O) Limbs from transgenic embryos carrying *lacZ* driven by the *Shh* promoter and either 1.7-kb ZRS (N) or the truncated DelB (O). The proximal extent of expression is marked in all cases by a white arrow.

Transgenic assays gauge regulatory activity of enhancers outside the normal chromosomal context and in close proximity to a promoter. In order to understand the full regulatory capacity, the ZRS was assayed in its native context by targeting mutations to this locus in embryonic stem cells (ESCs) to modify the endogenous genomic sequence. The targeted alleles were designed to include the *lacZ* reporter gene inserted downstream to monitor the regulatory activity of the mutated ZRS from a short range (Fig. 3A). Three targeting constructs were made (supplementary material Fig. S1A,B). Firstly, a construct that deleted the 3′ end fragment (region deleted in the DelB transgenic) was targeted and the mutant mice were designated ZRS^3′del+LACZ/+^ (Fig. 3A). The second construct (Fig. 3A) was similar but with the single remaining ETS1/GABPα binding site inactivated and this line was designated ZRS^ETS+LACZ/+^. The third was the control construct (Fig. 3A) in which the ZRS was left intact but the reporter gene was placed downstream and this line was designated ZRS^wt+LACZ/+^.

Initially, the *lacZ* expression pattern (Fig. 3B-E) was analysed in the different mouse lines. Two different mouse lines carrying the ZRS^3′del+LACZ^-targeted locus (designated ZRS^3′del+LACZ20^ and ZRS^3′del+LACZ32^) were generated to ensure that any phenotype detected was due to the targeted mutation, and subsequent analysis on both lines showed no detectable differences in the limb bud phenotype. (In those instances in which no specific line number is stated, the line shown is ZRS^3′del+LACZ20^.) Expression in ZRS^3′del+LACZ20/+^ was compared with that of the ZRS^wt+LACZ12^ line (shown in Fig. 3B-E). Both spatial and temporal expression were accurately regulated in the ZRS^3′del+LACZ/+^ lines compared with ZRS^wt+LACZ/+^, initiating expression at the appropriate stage, ∼E10.5 (Fig. 3B,C), in both fore and hind limb and continuing to E11.5 (Fig. 3D,E). Expression of *lacZ* regulated by ZRS^3′del+LACZ/+^ did, however, show a decrease in width compared with ZRS^wt+LACZ^ (compare Fig. 2E with 2D) presumably due to the differences in number of ETS binding sites (Lettice et al., 2012). Levels of *lacZ* mRNA in both the ZRS^3′del+LACZ/+^ lines were compared with the ZRS^wt+LACZ^ line by qRT-PCR (Fig. 3H). Limbs from 11 ZRS^wt+LACZ12^, eight ZRS^3′del+LACZ20/+^ and 14 ZRS^3′del+LACZ32/+^ were analysed (Fig. 3H). The short-range activity was further analysed by *in situ* hybridisation. As the selection cassette used to make the ESC lines also contains a PGK-neo^R^ gene, probes to both neo^R^ and *lacZ* were used and showed no detectable differences in expression levels (Fig. 3J-M). These analyses showed that levels of expression, monitoring short-range transcriptional activity, in the limb bud are similar whether the ZRS is intact or is lacking a significant portion of the conserved 3′ end.

The ESC clones carrying either the ZRS^ETS+LACZ^ or the ZRS^3′del+LACZ32^ allele were injected into tetraploid blastocysts to make high percentage ESC-derived embryos (Fig. 3F,G) (Nagy et al., 1993). In these embryos, the ZRS allele carrying the ETS mutation produced no detectable *lacZ* expression (*n*=5) in the limb whereas the ZRS^3′del+lacZ^ allele expressed in the expected pattern at the posterior margin of the limb (*n*=6) (*lacZ* expression at other *Shh* expression sites in both alleles was similar; data not shown). The specific, inactivating ETS mutation shows that expression of the nearby reporter genes is due directly to the ZRS element and not to cryptic regulatory activity acting outside the ZRS (i.e. secondary or shadow enhancers) (Perry et al., 2010; Frankel et al., 2010).

**Fig. 4.**
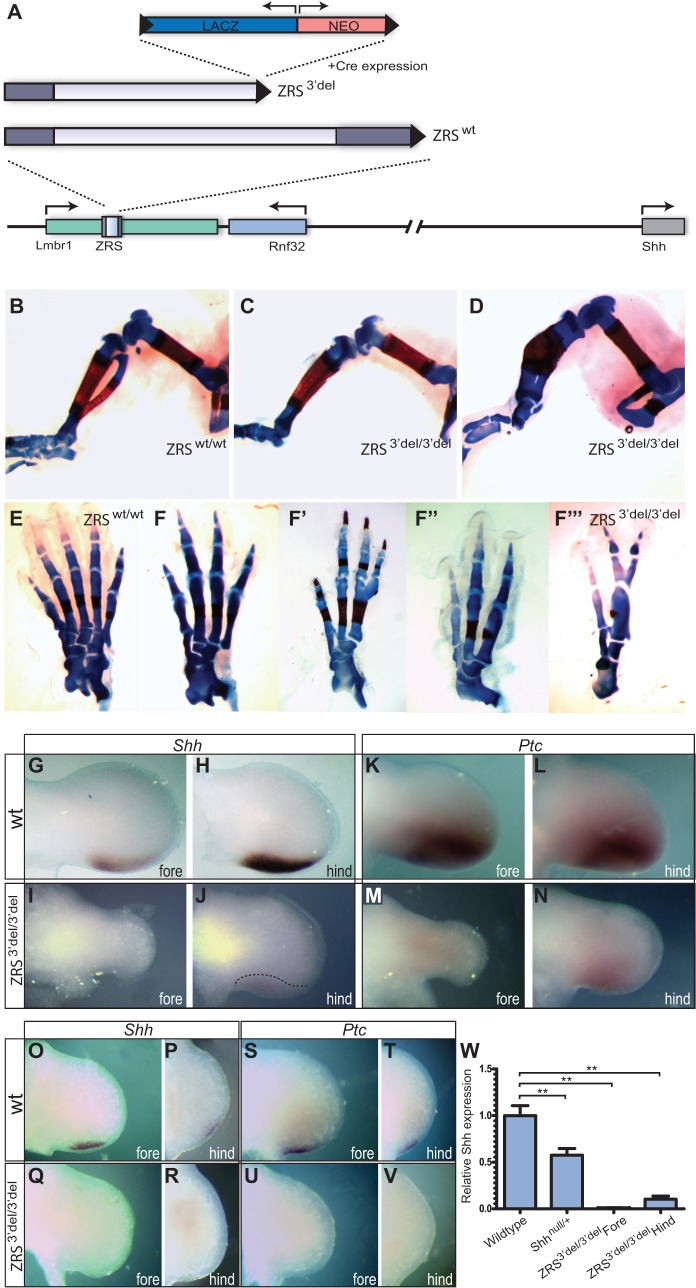
**Analysis of limb development in the ZRS deletion mutant.** (A) Mutations made in the ZRS after removing the *lacZ* reporter and the Neomycin^R^ (NEO) selectable marker genes with Cre recombinase leaving behind a single LoxP site (black triangle). (B-V) All of the images shown are taken from ZRS^3′del20^ derived from the ZRS^3′del+LACZ20^ line. (B-D) The long bones of the hind limbs are shown in ZRS^wt/wt^ (B) and ZRS^3′del/3′del^ (C,D). (E-F‴) The range of digit number that forms in the hind limb in the ZRS^3′del/3′del^ mutants (F-F‴) compared with the wild-type pattern produced by ZRS^wt/wt^ (E). (G-N) Expression analysis in E11.5 embryos of *Shh* (G-J) and *Ptc* (K-N) in wild-type (wt) mice and ZRS^3′del/3′del^ fore and hind limbs as indicated. For *Shh* expression, *n*=15 wt and 10 mutants and for *Ptc* expression *n*=16 wt and 6 mutants. Note the low expression of *Shh* but in a normal pattern (indicated by the dotted line) (J) and the lower levels of *Ptc* (N) compared with wt. (O-V) At an earlier stage, E10.5, expression of *Shh* (O,P) and *Ptc* (S,T) is detected in wt limbs. However, expression of both *Shh* (Q,R) and *Ptc* (U,V) is undetectable in ZRS^3′del/3′del^ limbs. For *Shh* expression, *n*=14 wt and 12 mutants and for *Ptc* expression *n*=9 wt and 5 mutants. (W) Levels of *Shh* expressed in the individual limb bud pairs of wild-type, heterozygous (*Shh*^null/+^) and mutant (ZRS^3′del/3′del^) fore and hind limb buds analysed by qRT-PCR. The mean±s.e.m. is plotted. These values were subjected to a non parametric Mann–Whitney U-test (**P<0.01).

The ZRS^3′del+LACZ/+^ and the ZRS^wt+LACZ/+^ mice were intercrossed and the homozygous mice showed limb phenotypes similar to those resulting from the knockout of the *Shh* gene itself (Chiang et al., 2001) and the full ZRS knockout (Sagai et al., 2005), with long bone abnormalities and severe distal truncations in both sets of limbs (Fig. 3I). These limb defects were presumably due to regulatory interference by the cassette containing the *lacZ* and neomycin resistance (*neo*^R^) gene (Fig. 3A). This regulatory interference was eliminated by the recombination of the surrounding loxP sites (Fig. 4A) in both the ZRS^3′del+LACZ/+^ and ZRS^wt+LACZ/+^ mice and these lines were designated ZRS^3′del/+^ and ZRS^wt/+^, respectively (Fig. 4A).

The ZRS^wt/+^and the ZRS^3′del/+^ mice were backcrossed to make homozygous mutant mice. The ZRS^wt/wt^ embryos had normal fore and hind limbs (Fig. 4B,E). The ZRS^3′del/3′del^ embryos, by contrast, consistently showed limb deformities. The forelimbs were severely affected, presenting with severe truncations similar to the *Shh* loss-of-function alleles (Chiang et al., 2001; Sagai et al., 2005) (data not shown). The hind limbs, however, presented with a unique series of digits forming in an incomplete pattern (Fig. 4F-F^‴^, Table 2). The range of hind limb phenotypes included thickening, shortening and often fusions of the tibia and fibula (Fig. 4C,D), loss or fusion of tarsals, loss of metatarsals often showing fusions and digits ranging from two to four (Fig. 4F-F^‴^). The ZRS^3′del^ crossed to the *Shh* null mouse (*Shh*^null/+^) results in ZRS^3′del/+^;*Shh*^null/+^ embryos with a similar range of limb deficiency phenotypes (Table 2). This genetic non-complementation confirms that the ZRS deletion mutation is operating through the *Shh* gene.

**Table 2.**
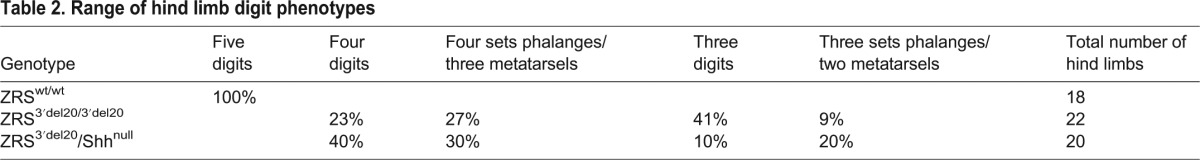
**Range of hind limb digit phenotypes**

The ZRS 3′ deletion is compromised at the level of normal long-range distance regulation, raising questions about the ability of the deletion mutations to recognise the *Shh* promoter. We argue that both the ZRS and the 3′ deletion mutation are promiscuous in their enhancer activity showing a capacity to recognise heterologous promoters (the β-globin promoter driving β-gal and the PGK promoters driving Neo^R^ in Fig. 3J-M). To examine specifically whether the ZRS 3′ deletion could recognise the *Shh* promoter, we made constructs containing the *Shh* promoter linked to the *lacZ* gene. This promoter construct contains the conserved 375 bp from the translational start codon extending 5′ to a non-conserved region of simple repeat. The expression of this construct, controlled by either the full-length 1.7 kb or the DelB fragment, was examined and both are capable of driving specific fore and hind limb expression (Fig. 3N,O) to a similar efficiency (Table 1). The construct in the absence of an added enhancer showed no consistent embryonic expression (Table 1). The 3′ domain of the ZRS does not appear to be required for recognition of the *Shh* promoter.

At E11.5, by *in situ* hybridisation, we show that *Shh* is normally expressed at the posterior margin of the limb (Fig. 4G,H) and *Ptc* (*Ptch1* – Mouse Genome Informatics), a sensitive readout for *Shh* activity (Marigo et al., 1996), is expressed deep into the middle of the limb bud (Fig. 4K,L). In the ZRS^3′del/3′del^ embryonic forelimbs at E11.5, the expression of both *Shh* (Fig. 4I) and *Ptc* (Fig. 4M) is undetectable. In the hind limbs, expression of *Shh* is low but detectable (Fig. 4J) and *Ptc* is considerably reduced (Fig. 4N). At E10.5, when *Shh* and *Ptc* are first detectable in both fore (Fig. 4O,S) and hind limbs (Fig. 4P,T), neither are detected in either set of limb buds in the ZRS^3′del/3′del^ embryo (Fig. 4Q,R,U,V), suggesting that there is a lag in the emergence of SHH activity. Expression levels of *Shh* were then analysed on pairs of limb buds from individual embryos at E11.5 by qRT-PCR and the mean of the individual pairs plotted (Fig. 4W; individual pairs of limbs from five wild-type and six mutant embryos were analysed). In forelimb, the levels of *Shh* mRNA was very low (<0.03% that of the wild type). In the hind limb, the levels are ∼10% of wild-type levels (Fig. 4W) showing that a surprisingly low level of *Shh* expression is necessary to initiate digit number specification. To establish a range in which *Shh* functions, we analysed levels of *Shh* expression in the heterozygous *Shh*^null/+^ mouse, which has normal legs (individual limb bud pairs from 13 wild type and 14 heterozygotes were analysed). As expected, *Shh* expression in limb buds from the *Shh^null^*^/+^ embryos was about half of wild-type *Shh* levels (55%, Fig. 4W) revealing that a dynamic range of *Shh* levels act to regulate digit number. Hence, in the hind limbs, digit 1 forms in the absence of *Shh* expression (Chiang et al., 2001), whereas at low levels (∼10% of wild-type levels), up to four well-formed digits develop and then by approximately half the normal levels the limbs have the wild-type complement. Therefore, formation of five digits in the hind limbs occurs at a *Shh* level between 10 and 55% of wild type, indicating that a significant buffering capacity exists for producing digits and that the upper limit for digit formation is set at five.

### 3′ deletion of the ZRS mediates chromosomal conformational changes

**Fig. 5.**
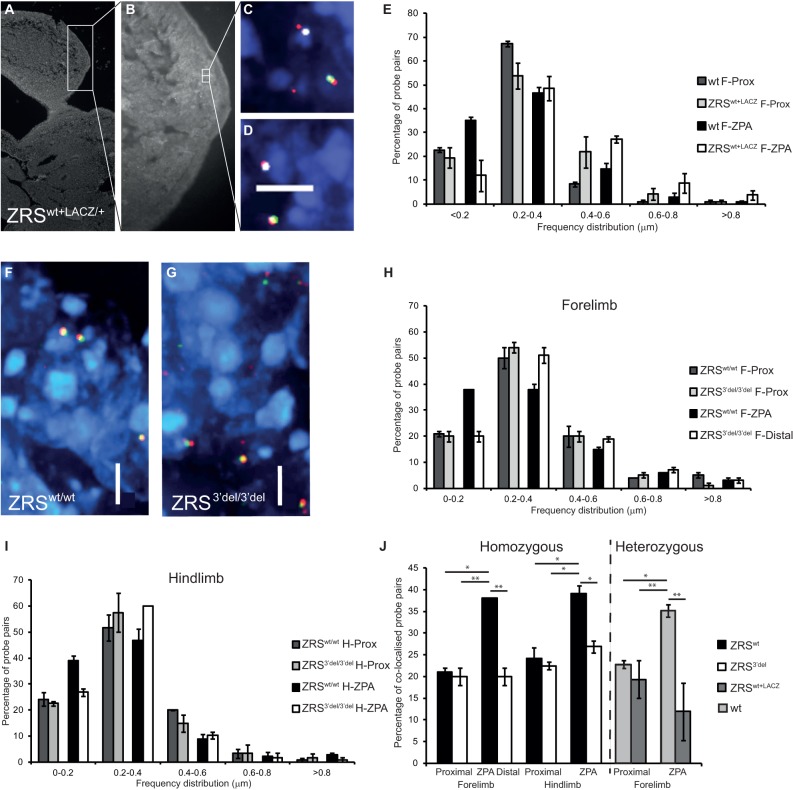
**Analysis of chromosome conformation by FISH.** (A,B) Low magnification image of a DAPI-stained section from a ZRS^wt+LACZ/+^ embryo (A) at E11.5 and a close up of the posterior limb bud, with the ZPA highlighted by staining for the SHH protein (B). (C,D) Four-colour FISH images showing two probe pairs from within the ZPA stained for *Shh* (red), the ZRS (green) and the *lacZ* reporter (white). The *lacZ* staining highlights the ZRS^wt+LACZ^ allele. (E) Graph of the distribution of interprobe distances between *Shh* and ZRS in the wild-type allele and the ZRS^wt+LACZ^ allele in the proximal limb bud and in the ZPA of a ZRS^wt+LACZ/+^ embryo. (F,G) Three-colour FISH images showing probe pairs in the ZPA from ZRS^wt/wt^ (F) and ZRS^3′del/3′del^ (G) embryos (*Shh* stained red and ZRS with green). (H,I) Distribution of interprobe distances between *Shh* and the ZRS in the proximal and distal fore limb (H) and hind limb (I) buds of ZRS^wt/wt^ and ZRS^3′del/3′del^ embryos. (J) Summary of the measurements and comparison of colocalised probe pairs in ZRS^wt/wt^, ZRS^3′del/3′del^ and ZRS^wt+LACZ/+^. Significant comparisons are indicated (*P<0.05, **P<0.01 compared with the relevant wild-type ZPA sample). Significantly greater Shh-ZRS colocalisation is identified in the ZPA of fore limb and hind limb in ZRS^wt^ compared with ZRS^3′del^, ZRS^wt+LACZ^ and proximal wild type. Loss of colocalisation at the *lacZ* allele in distal posterior nuclei corresponds to an increased proportion of probe pair distances greater than 400 nm. No detectable difference in probe colocalisation occurs in the proximal nuclei. Between 100 and 175 loci were measured for each tissue and probe pair. Tables of the results of the statistical tests are shown in supplementary material Tables S3-S6. F-Prox, fore limb proximal; F-ZPA, fore limb ZPA; F-Distal, fore limb distal posterior region; H-prox, hind limb proximal; H-ZPA, hind limb ZPA; Wt, wild type. Scale bars: 5 µm.

Amano et al. (2009) showed that the competence to express *Shh* in the limb bud is due to long-range enhancer-promoter interactions. To analyse changes to long-range chromosomal conformation due to the 3′ deletion of the ZRS enhancer, we used 3D-FISH (fluorescence *in situ* hybridisation to chromosomal DNA) (Chambeyron and Bickmore, 2004; Morey et al., 2007; van de Corput et al., 2012) using individual fosmid probes encompassing the ZRS and the *Shh* gene (supplementary material Table S2) or a cloned probe carrying the *lacZ*/Neo^R^ selection cassette. We chose to examine samples at E11.5 as *in situ* data shows that *Shh* is expressed in the hind limbs of ZRS^3′del/3′del^ at this stage, making it possible to compare active (in ZRS^3′del/3′del^) and inactive (in ZRS^wt+lacZ^) conformations at the same stage. Thus, E11.5 day embryos were sectioned and the region of the limb that expresses *Shh* (the ZPA) was determined by immunohistochemistry using an anti-*Shh* antibody raised against the non-signalling C-terminal portion of the protein (Fig. 5A,B). Alternate sections were used to highlight the *Shh*-expressing regions for FISH analysis.

Initially, the heterozygous E11.5 embryos carrying the ZRS^wt+lacZ^ allele were examined. In the cells analysed, we were able to distinguish the wild-type allele from the insertion allele (carrying the selection cassette and *lacZ*) using probes for *Shh*, the ZRS and *lacZ*/PGK-Neo^R^ (Fig. 5C,D). Distances were measured in cells in expressing (ZPA) and non-expressing (limb tissue proximal to the ZPA) regions of the limb and the distribution in 0.2-μm intervals was plotted (Fig. 5E). In the non-expressing tissue, the distance between the ZRS and the *Shh* gene was similar for each allele, particularly at the closest interval (<0.2 μm). In the ZPA, the conformation of the wild-type allele (WT F-ZPA, Fig. 5E) showed significantly closer association between the ZRS and the *Shh* gene than in non-expressing cells, in agreement with a previous report (Amano et al., 2009). In the insertion allele, however, the association was not significantly different from that in non-expressing cells (Fig. 5E), suggesting that although the ZRS is active at close range to drive *lacZ* expression, the insertion element inhibited long-range interactions. The insertion carries two basal promoters and presumably successfully competes for the ZRS enhancer activity. (*P*-values for the statistical analyses can be found in supplementary material Tables S3-S6.)

We next looked at the intact ZRS (ZRS^wt^) and the ZRS 3′ deletion (ZRS^3′del^) alleles (Fig. 5F,G) in which the selection cassettes (*lacZ* and PGK-neo) had been removed. The ZRS^wt^ allele showed a significant difference in ZRS and *Shh* colocalisation in expressing and non-expressing regions of the limb and was similar to the unmodified wild-type allele. The ZRS^3′del/3′del^, by contrast, showed no differences in the forelimb (Fig. 5H). Here, the comparison was between proximal non-expressing tissue and distal posterior cells in which *Shh* would normally be expected to be expressed. In the hind limbs, expressing tissue is still identifiable by the *Shh* antibody. Interestingly, measurements show that in the ZRS^3′del/3′del^ a mean of 27% of the nuclei show close proximity (<0.2 μm), significantly different from 39% determined for the ZRS^wt/wt^ (*P*=0.04) (Fig. 5I,J). In comparison with non-expressing tissue, which shows a mean of 22.5%, there may be a difference which correlates with the low levels of expression in the ZRS^3′del/3′del^; however, this association is not significant (Fig. 5J). (Tables of *P*-values can be found in supplementary material Tables S3-S6.) Thus, there is a correlation between expression levels of the *Shh* gene and the conformation of the regulatory domain. Deletion of the 3′ end of ZRS results in a reduction in the ability of the ZRS to undergo the chromosomal confirmation associated with gene activity.

## DISCUSSION

Insights into structure-function relationships of *cis*-regulatory elements are particularly important for understanding how development is programmed. We showed that the conserved DNA sequence that constitutes the ZRS highlights essential interacting regulatory elements. The ZRS sequence can be dissected in transgenic assays into two domains: an active spatial domain that resides within the 5′ half and an apparently dispensable 3′ half that shows no independent spatial activity. However, transgenic analysis supports the notion that the seemingly superfluous sequence encodes information that influences the spatial activity and that integration of information incorporated throughout the ZRS is responsible for full activity. These data fit an enhanceosome model in which the ZRS is organised into different regulatory components that act as a cooperative unit (Panne, 2008).

The function of the 3′ region of the ZRS became obvious after making the mutations at the endogenous locus. Comparison of the intact and 3′-deleted ZRS showed that both are capable of regulating spatial expression at similar levels from a close range but the 3′ end sequence is required to facilitate long-range activity. This inability to drive *Shh* expression efficiently is not due to lack of recognition of the gene-specific promoter. Although the ZRS^3′del^ allele is inactive at a long range in the fore limbs, in the hind limbs there is low but detectable expression of *Shh*, indicating a reduction in long-range activity. An element dedicated to long-range activity has been identified in *Drosophila* (Swanson et al., 2010); we suggest, here, that there is no specific element within the ZRS but rather that long-range activity is due to supplementary regulatory information that boosts the regulatory response. Enhancers are often considered to be binary switches turning genes on and off (Fiering et al., 2000); but, clearly, in the case of the ZRS, an additional set of regulatory instructions is required to meet the challenge of regulating from a distance.

A number of models for how distal regulatory elements communicate with their target promoters have been suggested and the simplest model with the most supporting evidence is of a direct physical interaction brought about by chromatin looping. We used 3D-FISH to examine the colocalisation of the ZRS and the *Shh* promoter and showed that the reduction in long-range activity mediated by the deletion of the 3′ half of the ZRS is reflected in the chromosomal conformation. We found a strong correlation between ZRS-promoter colocalisation and expression activity in the limbs in the ZRS alleles containing the interfering selection cassette (ZRS^wt+lacZ^ and ZRS^3′del+lacZ^) and the allele with the 3′ end deletion (ZRS^3′del^). Our data suggest a direct relationship between *Shh* gene expression in the limb bud and chromosomal conformation. As a consequence, we argue that at E11.5 the inactivity of the ZRS^3′del^ in the forelimb and low level of activity in the hind limb is due to an inability to generate the conformation required for gene activity. However, this may be embryonic stage specific. A report (Amano et al., 2009) analysing earlier staged embryos (E10.5) showed that in mice with a complete deletion of the ZRS the presence of the enhancer was not required for the interaction of this region with the *Shh* gene. Therefore, a complex picture is developing in which the ZRS at the earliest stages of limb bud development is driven to interact with the *Shh* gene by as-yet-unidentified elements, but this task is subsequently acquired by the ZRS itself by E11.5. We suggest that the 3′ half of the ZRS operates by boosting activity within this mechanism that mediates these enhancer-promoter interactions.

The attenuated expression produced by the ZRS 3′ end deletion revealed an unexpected variability inherent in regulating digit number. The variation in the limb phenotype ranged from a *Shh* null phenotype in the fore limbs to two to four digits with deletions and fusions of tarsals and metatarsals in the hind limbs. Two parameters of expression that are predicted to be important for SHH morphogen function, i.e. levels and temporal extent of exposure (Yang et al., 1997; Ahn and Joyner, 2004; Harfe et al., 2004), were both affected by the deletion. In addition, the fore limbs showed a greater sensitivity than the hind limbs to regulatory perturbations created by the deletion. The fore and hind limbs are serial homologues that were derived in evolution by duplication from a single set of primordial paired appendages (Shubin et al., 1997). Although fore limbs are structurally distinct from hind limbs in all tetrapods, the pairs share a number of defining skeletal characteristics, one of which is the basic number of digits that form. The early developmental programme that establishes the *Shh* expression in the ZPA to specify digit number is integrated into both sets of limbs. The regulation of this programme, as shown here, does not operate equivalently in the fore and the hind limbs. The 3′ end of the ZRS provides a robust long-range response that ensures that SHH signalling levels are sufficient in both sets of limb buds to promote normal limb patterning.

Buffering of developmental processes against environmental perturbations or mutations was described as canalisation (Waddington, 1942), which has evolved in order to stabilise a phenotype (Gibson and Wagner, 2000). This was shown for some *Drosophila* regulators; for example, the *eve* stripe 2 enhancer (Ludwig et al., 2011). The minimal stripe 2 enhancer (480 bp) produces viable offspring; however, surrounding conserved sequences (encompassing 800 bp) are required to buffer against genetic and environmental perturbations. Here, a region of the ZRS provides robustness to *Shh* limb expression, which ensures an invariant phenotype during limb development, a process that provides developmental stability (Jamniczky et al., 2010). High sequence conservation of the ZRS in vertebrates (Lettice et al., 2003) and the limb pattern based on a limit of five digits (Long and Gordon, 2004) is recurrent in land-based tetrapods, suggesting that the phenotype was stabilised in tetrapod evolution and is fundamental to the basic structure of vertebrate limbs. We argue that the robustness assayed here evolved to overcome the intrinsic problems of a long-range regulator and provides a mechanism that adds stability to the limb phenotype in tetrapods.

## MATERIALS AND METHODS

### Transgenic constructs

The full-length ZRS transgenic construct was previously described (Lettice et al., 2003), whereas the inserts for the other constructs were generated by PCR and subcloned into the p1230 vector (Yee and Rigby, 1993) containing the *lacZ* reporter gene and a β-globin minimal promoter. The *Shh* promoter constructs were made by cloning the 1.7-kb or DelB ZRS fragment upstream of a *Shh* promoter that had been generated by PCR. (Primer sequences are in supplementary material Table S1.)

### Production of constructs for ESC gene targeting

A 7-kb genomic fragment was retrieved from PAC 542-N10 from the RCPI21 library (Osoegawa et al., 2000), corresponding to nucleotides 29, 636, 979 to 29, 643, 992 from mouse chromosome 5 of assembly NCBIM37 (www.ensembl.org). A mini-targeting vector was constructed, based on PL452 (Liu et al., 2003) and the whole construct assembled by recombineering (Liu et al., 2003). Details of the constructs and resulting alleles are shown in supplementary material Fig. S1. E14Tg2a ESCs were targeted and each line was screened using primer sets 1f and 1r and 2f and 2r (supplementary material Fig. S1). Correctly targeted ESC clones were microinjected into C57BL6/J embryos to make chimaeras (Dorin et al., 1992). Tetraploid complementation embryos were produced by electrofusion (Nagy et al., 1993) to make entirely ESC-derived embryos. Cre-mediated recombination was performed by crossing to a line carrying a pCAGGS-Cre recombinase gene (Araki et al., 1995).

### *lacZ* expression analysis, skeletal staining and *in situ* hybridisation

Embryos were analysed for *lacZ* expression at E10.5 or E11.5 by staining for β-gal activity as previously described (Lettice et al., 2008). Skeletal preparations from E17.5 fetuses were stained simultaneously with Alizarin Red and Alcian Blue (Nagy et al., 2009a,b). Whole-mount *in situ* hybridisation was performed as previously described (Hecksher-Sorensen et al., 1998).

### RNA extraction for qRT-PCR analysis

The limb buds from individual E11.5 embryos were removed and pairs of fore limbs and hind limbs were separately snap frozen, genotyped and RNA extracted using TriReagent (Sigma). Multiplex qPCR assays were performed on a LightCycler480 Real Time PCR System (Roche). Universal ProbeLibrary Reference Gene Assays (Roche) were included such that each sample contained the internal control *Gapdh* reference gene probe and primers in addition to the target probe (#32 for *Shh* and #18 for *lacZ* from the Universal Probe Library) and primers. Each assay was run in triplicate and the mean Cp and the target/reference ratios calculated by the LightCycler480 software (Release 1.5.0). To correct for the variation between litters, the mean wild-type value for each litter was calculated and the individual relative *Shh* RNA values for both wild type and mutants was divided by the wild-type litter mean. This produces a relative *Shh* RNA value that is normalised to the wild-type litter mean. These values were subjected to a non-parametric Mann–Whitney U-test and plotted ±s.e.m. using GraphPad Prism 5. Values for *lacZ* expression were normalised to the levels of expression in ZRS^wt^.

### Mouse embryo sectioning and 3D DNA FISH

E11.5 embryos were fixed in 4% formaldehyde, paraffin embedded and 6-µm-thick sections cut. Alternate slides carrying sections through the limb buds were stained with an antibody raised against the C-terminus of SHH (Abcam, ab86462; 1:100) to identify the ZPA and the adjacent slides subjected to FISH. Fosmid clones (supplementary material Table S2) were labelled with digoxigenin-11-dUTP, biotin-16-dUTP or directly labelled with green dUTP as previously described (Morey et al., 2007). The probe for the *lacZ*-carrying chromosome contained both the *lacZ* and the PGK neo^R^ genes and was labelled with biotin-16-dUTP. For three-colour FISH, 200 ng of biotin- and digoxigenin-labelled fosmid probes were used per slide, whereas for four-colour FISH 300 ng of biotin-, digoxigenin- and directly labelled fosmid probes were used per slide, with 20-30 µg of mouse Cot1 DNA (Invitrogen) and 10 µg salmon sperm DNA. Hybridisations were performed as previously described (Chambeyron and Bickmore, 2004; Morey et al., 2007).

For 3D analysis of tissue sections, slides were imaged with a Hamamatsu Orca AG CCD camera (Hamamatsu Photonics), Zeiss Axioplan II fluorescence microscope with Plan-neofluor or Plan Apochromat objectives, a Lumen 200 W metal halide light source (Prior Scientific Instruments) and Chroma #89014ET single excitation and emission filters (Chroma Technology Corp.). Images were deconvolved using a calculated PSF with the constrained iterative algorithm of Volocity (PerkinElmer). Image analysis was carried out using the Quantitation module of Volocity (PerkinElmer). For 3D-FISH, 100-175 loci were measured for each tissue and for each probe combination and subjected to Fisher's exact test.

## Supplementary Material

Supplementary Material
