# Supplementary material for: Development of five digits is controlled by a bipartite long-range cis-regulator
Source: Development. 2014 Apr;141(8):1715–25. doi: 10.1242/dev.095430 (PMC3978833; doi:10.1242/dev.095430)
Supplement: Supplementary Material [file supp_141_8_1715__index.html]

Supplementary Material 

# Development of five digits is controlled by a bipartite long-range *cis*-regulator

## DEV095430 Supplementary Material

**Files in this Data Supplement:**

- **Supplementary Material**
