# Supplementary material for: Development of five digits is controlled by a bipartite long-range cis-regulator
Source: Development. 2014 Apr;141(8):1715–25. doi: 10.1242/dev.095430 (PMC3978833; doi:10.1242/dev.095430)
Supplement: Supplementary Material [file supp_141.8.1715_DEV095430.pdf]

Fig. S1. Details of the ZRS targeting constructs and representation of the correctly inserted into the locus before and after Cre-recombinase. (A) The three constructs which were targeted into the endogenous ZRS (shown as a grey rectangle in the bottom line, with HindIII (H) sites at either end). These constructs comprise a 7kb region of genomic DNA flanked by NotI and SalI restriction sites (N and Sl respectively), carrying a bacterial LacZ gene driven by a minimal β-globin promoter (inserted into a SmaI site (S)) and a Neomycin resistance (neo) resistance cassette driven by a PGK promoter. The mutated ETS site in ZRS^{ETS} is indicated by the black rectangle. (B) The loci after correct targeting, which was screened for by PCR using primers 1f and 1r and 2f and 2r across homology arms 1 and 2 respectively. Mice carrying these chromosomes were genotyped for the presence of lacZ using primers Lf and Lr and for the presence of $neomycin^R$ using Nf and Nr. The unmodified wildtype chromosome was screened for using Wf and R. (C) The result of cre mediated excision of the lacZ and $neomycin^R$ cassettes which leaves only a single loxP site. Genotyping of mice with Wf and R or Df and R allows the modified and wildtype chromosomes to be distinguished because of the size difference created by the remaining lox site.

Lettice et al., Figure S1

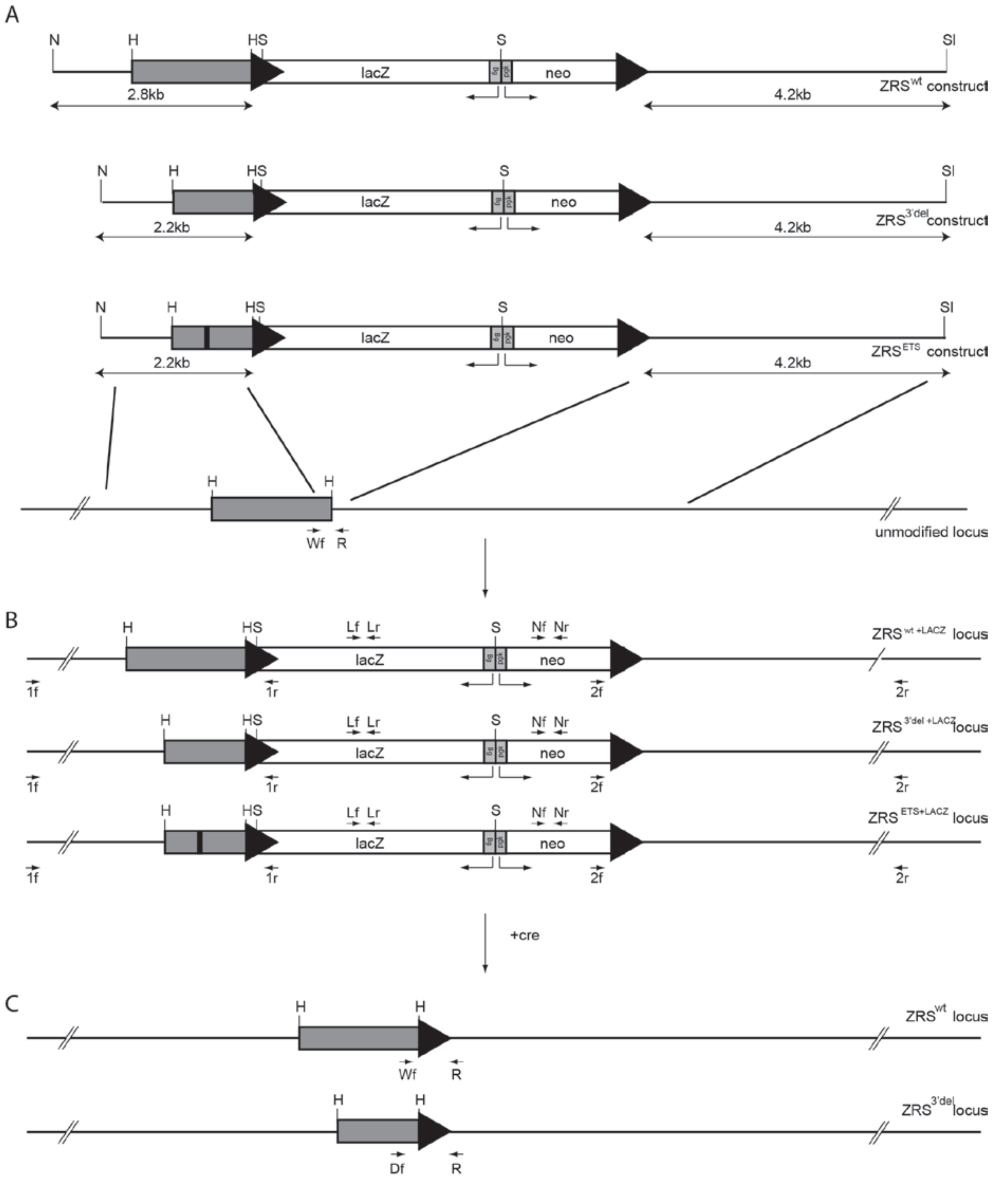

Table S1. Primer sequences

Constructs		
DelA	F- GAATTCTCAAGTATAAGAGAACATAG<br>R- gatcat**AAGCTT**AACAAGCAAAAATAAGAAAG	
DelB	F- GAATTCTCAAGTATAAGAGAACATAG<br>R- gatcat**AAGCTT**TAAGATGGAGGCCTGAGAC	
DelC	F- GAATTCTCAAGTATAAGAGAACATAG<br>R- gatcat**AAGCTT**GTCATCTGGTCATAAAATACAG	
DelD	F- GAATTCTCAAGTATAAGAGAACATAG<br>R- gatcat**AAGCTT**CATATTAAAACGTACTTAGTTC	
3' END	F- gatcat**AAGCTT**TTTCTATCCTGTGTCACAGTTTG<br>R- TACTAAGAAGGTGGCAACAGAC	
Core	F- gatcat**AAGCTT**AGTCCTGGCATAAAACTTAAC<br>R- gatcat**AAGCTT**TAAGATGGAGGCCTGAGAC	
Mutagenise Hx	F- GCTTGTTTTTTTTGCCACTAATGATCCATAAATTGTTGGAAATGAG<br>R- CTCATTTCCAACAATTTATGGATCATTAGTGGCAAAAAAAACAAGC	
Mutagenise M100081	F- GTGACCTTGTACTGTGTTTTATGACCAGATGACTTTTCCCCTCAG<br>R- CTGAGGGGAAAAGTCATCTGGTCATAAAACACAGTACAAGGTCAC	
-ETS	F- GCACAAAATCTGAGGTCACTTGAACTCTTAATTAGTTGCACTGACCAGG<br>R- CCTGGTCAGTGCAACTAATTAAGAGTTCAAGTGACCTCAGATTTTGTGC	
Mutagenise REP3A	F- GCTTGTTTTTTTTGCCACCAGTGATCCATAAATTGTTGGAAATGAGC<br>R- GCTCATTTCCAACAATTTATGGATCACTGGTGGCAAAAAAAACAAGC	
Mutagenise REP3B	F- GCTTGTTTTTTTTGCCACAATTGATCCATAAATTGTTGGAAATGAGC<br>R- GCTCATTTCCAACAATTTATGGATCAATTGTGGCAAAAAAAACAAGC	
Flip80<br>Region flipped is underlined	Arm 1 gatcat**AAGCTT**TTTAATGCCTATCATTTGATTTGAAGTC and<br>ATAAATTGTTGGAAATGAGCGATTCAGGAAGTGCTGCTTAGTGTTAGTGGCAAATGCGCTAACAAGCAA<br>AAATAATGAAAGAATC<br>Arm 2<br>AAGCAGCACTTCCTGAATCGCTCATTTCCAACAATTTATGGATCATCAGTGGCAAAAAAACTCAGTCT<br>GGTTCTGCTG<br>and gatcat**AAGCTT**ACATAGCAACAGTTAGTGAGATATG	
Flip80 +Hx	Arm 1 gatcat**AAGCTT**TTTAATGCCTATCATTTGATTTGAAGTC and<br>ATAAATTGTTGGAAATGAGCGATTCAGGAAGTGCTGCTTAGTGTTAGTGGCAAATGCGCTAACAAGCAA<br>AAATAATGAAAGAATC<br>Arm 2<br>AAGCAGCACTTCCTGAATCGCTCATTTCCAACAATTTATGGATCATTAGTGGCAAAAAAACTCAGTCT<br>GGTTCTGCTG<br>and gatcat**AAGCTT**ACATAGCAACAGTTAGTGAGATATG	
Flip80+Rep3A	Arm 1 gatcat**AAGCTT**TTTAATGCCTATCATTTGATTTGAAGTC and	

	ATAAATTGTTGGAAATGAGCGATTCAGGAAGTGCTGCTTAGTGTTAGTGGCAAATGCGCTAACAAGCAAAAATAATGAAAGAATC<br>Arm 2<br>AAGCAGCACTTCCTGAATCGCTCATTTCCAACAATTTATGGATCACTGGTGGCAAAAAAACTCAGTCTGGTTCTGCTG<br>and gatcat**AAGCTT**ACATAGCAACAGTTAGTGAGATATG	
FLIP49<br>Region flipped is underlined	Arm 1 gatcat**AAGCTT**TTTAATGCCTATCATTTGATTTGAAGTC and<br>CGTTCATTGGATTCTTTCATTATTTTTGCTTGTTTTTTTTGCCACTGATCTCATGGAGTCCCAGGC<br>Arm 2 AACAAGCAAAAATAATGAAAGAATCCAATGAACGGATCCATAAATTGTTGGAAATG<br>and gatcat**AAGCTT**ACATAGCAACAGTTAGTGAGATATG	
FLIP49+REP3A	Arm 1 gatcat**AAGCTT**TTTAATGCCTATCATTTGATTTGAAGTC and<br>CGTTCATTGGATTCTTTCATTATTTTTGCTTGTTTTTTTTGCCACCTGTCTCATGGAGTCCCAGGC<br>Arm 2 AACAAGCAAAAATAATGAAAGAATCCAATGAACGGATCCATAAATTGTTGGAAATG<br>and gatcat**AAGCTT**ACATAGCAACAGTTAGTGAGATATG	
REP5	Arm1- GAATTCTCAAGTATAAGAGAACATAG and<br>CATTATTTTTGCTTGTTTTTTTTGCCTGCAGTGATCCATAAATTGTTGGAAATGAGC<br>Arm 2- GCTCATTTCCAACAATTTATGGATCACTGCAGGCAAAAAAAACAAGCAAAAATAATG and<br>TACTAAGAAGGTGGCAACAGAC	
Del41	Arm 1 gatcat**AAGCTT**TTTAATGCCTATCATTTGATTTGAAGTC and<br>GTACTGTATTTTATGACCAGATGACAGAGAGTAGGAAGTCCAGCCTGG<br>Arm 2 CCAGGCTGGACTTCCTACTCTCTGTCATCTGGTCATAAAATACAGTAC<br>and gatcat**AAGCTT**ACATAGCAACAGTTAGTGAGATATG	
Shh promoter<br>ATG is underlined	F- gatcat**GTCGAC**TAAACTGGAAGCCTCAGGTG<br>R- gatcat**GGTACC**ATCTCGTCCGCGGAACCTGAG	
ES cell targeting screening		
Arm 1	1f- ATGGCACTGAAGTGTTGACTG<br>1r- CACTGCATTCTAGTTGTGG	
Arm 2	2f- ATCGCATTGTCTGAGTAGGTG<br>2r- GACCAATAGTACTCCAAGCAAG	
Genotyping		Allele size
lacZ	LF- CAACTTAATCGCCTTGCAGCAC<br>LR- CGCTGATTTGTGTAGTCGGTT	+lacZ - 550bp
Neo	NF- TGTTCCGGCTGTCAGCGCAG<br>NR- GATATTCGGCAAGCAGGCATC	+neo - 480bp
ZRS^{Del}	DF- CCAAGCAACATGACAGCAC<br>R- CACACACGGATACTAAGACGG	Wt- 950bp<br>ZRS^{Del} – 440bp
ZRS^{Wt}	WF- GGAATGCATGCAGGAACTCAG<br>R- CACACACGGATACTAAGACGG	Wt- 290 bp<br>ZRS^{Wt} – 380bp

qRT-PCR		
Shh	F- ACCCCGACATCATATTTAAGGA<br>R- TTAACTTGTCTTTGCACCTCTGA	
B-gal	F- ATGGATGAGCAGACGATGG<br>R- CGGCGTTAAAGTTGTTCTGC	

Table S2. Fosmid Probes

Region	Whitehead (Sanger) Name	Ensemble name	Coordinates Start	End	Size (bp)
Shh	WI1-574O18	G135P64333A4	28754458	28795879	41421
ZRS	WI1-1047E14	G135P600929F6	29611727	29653695	41968

Names are Ensembl (r 45) (http://jun2007.archive.ensembl.org/Mus_musculus/index.html). Mouse genome assembly number: NCBI m37.

Table S3. Co-localisation frequency of the *Shh* probe with the ZRS and *LacZ* reporter construct probes for E11.5 limb bud sections

Limb region	***Shh*-ZRS (847 kb)**	***Shh*-LacZ (847 kb)**	Probe pairs	ZPA	Proximal
	Co-localisation frequency (%)			Co-localisation frequency (%)	
**ZPA**	35.1	11.9	***Shh*-ZRS**	35.1	22.7
**Proximal**	22.7	19.3	***Shh*-LacZ**	11.9	19.3
	$p = 0.04$	$p = 0.29$		$p = 0.0002$	$p = 0.63$

Statistical analysis of data for Figure 5J- *p*-values from Fisher's Exact Tests.

Table S4. Frequency of Shh-ZRS and *Shh-LacZ* probes separated by ≥ 400nm for E11.5 limb bud sections

Probe pairs	ZPA	Proximal
	Frequency (%) ≥ 400 nm	
***Shh*-ZRS (847 kb)**	18.3	11.1
***Shh*-LacZ (847 kb)**	39.5	30.5
	$p = 0.0011$	$p = 0.0012$

Statistical analysis of data for Figure 5J- *p*-values from Fisher's Exact Tests

Table S5. Co-localisation frequency of the *Shh* probe with the ZRS probe for E11.5 limb bud sections derived from the fore limbs of ZRS$^{wt/wt}$ and ZRS$^{3'del/3'del}$ homozygous embryos

Forelimb	***Shh*-ZRS in ZRS$^{wt/wt}$ (847 kb)**	***Shh*-ZRS in ZRS$^{3'del/3'del}$ (847 kb)**	Probe pairs	ZPA/ Distal posterior	Proximal
	Co-localisation frequency (%)			Co-localisation frequency (%)	
**ZPA/Distal posterior**	38	20	***Shh*-ZRS in ZRS$^{wt/wt}$**	38	21
**Proximal**	21	20	***Shh*-ZRS in ZRS$^{3'del/3'del}$**	20	20
	$p = 0.01$	$p = 1$		$p = 0.008$	$p = 1$

Statistical analysis of data for Figure 5J- *p*-values from Fisher's Exact Tests

Table S6. Co-localisation frequency of the *Shh* probe with the ZRS probe for E11.5 limb bud sections derived from the hind limbs of $ZRS^{wt/wt}$ and $ZRS^{3'del/3'del}$ homozygous embryos

**Hindlimb**	***Shh*-ZRS in $ZRS^{wt/wt}$ (847 kb)**	***Shh*-ZRS in $ZRS^{3'del/3'del}$ (847 kb)**	**Probe pairs**	**ZPA**	**Proximal**
	**Co-localisation frequency (%)**			**Co-localisation frequency (%)**	
**ZPA**	39.1	26.9	***Shh*-ZRS in $ZRS^{wt/wt}$**	39.1	24.2
**Proximal**	24.2	22.5	***Shh*-ZRS in $ZRS^{3'del/3'del}$**	26.9	22.5
	$p = 0.009$	$p = 0.45$		$p = 0.04$	$p = 0.88$

Statistical analysis of data for Figure 5J- *p*-values from Fisher's Exact Tests
